# Cyanobacteria-specific algicidal mechanism of bioinspired naphthoquinone derivative, NQ 2-0

**DOI:** 10.1038/s41598-018-29976-5

**Published:** 2018-08-02

**Authors:** Heon Woo Lee, Bum Soo Park, Jae-Hyoung Joo, Shailesh Kumar Patidar, Hye Jeong Choi, EonSeon Jin, Myung-Soo Han

**Affiliations:** 1 Department of Life Science, College of Natural Sciences, Hanyang University, Seoul, 04763 South Korea; 2 Marine Science Institute, University of Texas at Austin, Port Aransas, TX 78373 USA; 3 Research Institute for Natural Sciences, Hanyang University, Seoul, 04763 South Korea

## Abstract

To mitigate cyanobacterial blooms, the naphthoquinone derivative, NQ 2-0, which has selective algicidal activity against cyanobacteria, has been developed. However, due to a lack of information on its algicidal mechanisms, there are significant gaps in our understanding of how this substance is capable of selectively killing cyanobacteria. Here, we investigated the selective algicidal mechanisms of NQ 2-0 using target (*Microcystis aeruginosa*) and non-target (*Cyclotella* sp. and *Selenastrum capricornutum*) species. NQ 2-0 showed selective algicidal activity against only *M. aeruginosa*, and this activity was strongly light-dependent. This NQ compound has selectively reduced the oxygen evolution rate and photosystem II (PSII) efficiency of *M. aeruginosa* throughout blocking electron transfer from the photosynthetic electron transport system, and significantly (*p* ≤ 0.05) increased levels of reactive oxygen species (ROS), resulting in membrane damage through lipid peroxidation. In ultrastructural observations, thylakoid membranes were disintegrated within 12 h after NQ 2-0 treatment, and cytoplasmic vacuolation and disintegrated cellular membrane were observed at 24 h. These findings suggest that increased ROS levels following NQ 2-0 treatment may induce cell death. Interestingly, compared to non-target eukaryotic cells, *M. aeruginosa* showed relatively late antioxidant response to reduce the increased ROS level, this may enhance algicidal activity against this cyanobacterium.

## Introduction

Harmful cyanobacterial blooms cause deleterious problems in aquatic ecosystems, such as scum formation, hypoxia, deterioration of water quality, and toxin production, affecting the health of wildlife and humans^1^. Due to worldwide climate change and nutrient load-triggered eutrophication over recent decades, cyanobacterial blooms in freshwater represent a growing threat; these blooms have expanded worldwide and their frequency has also sharply increased^2,3^. In response to this, many researchers have attempted to develop physical, chemical, and biological techniques to mitigate harmful cyanobacterial blooms^4^. Among these, chemical techniques have been the most widely used due to their high efficiency and low economic cost, and a number of commercial chemical algicides, such as copper sulfate^5,6^, diuron^7^, and Phoslock^® ^^8^, have been developed. However, there are serious disadvantages associated with the use of these substances; they have nonselective toxicity, resulting in side effects on the aquatic ecosystem^9^.

Since the development of an effective and selective algicide was needed to overcome the disadvantages of the abovementioned techniques, bio-derived substances have received attention^10,11^. Naphthoquinone derivative 2-0 (NQ 2-0), which was developed using bioinspired naphthoquinone as a precursor, demonstrated rapid, effective, and selective algicidal activity against harmful cyanobacteria in previous laboratory experiments^12^; cyanobacterium *Microcystis*, *Dolichospermum*, *Aphanizomenon*, *Oscillatoria*, and *Nostoc* sp. were eliminated by NQ2-0 by up to 90% within 2 days. Moreover, in a large scale (30 ton) mesocosm experiment, although this substance showed consistently high algicidal activity against target cyanobacterial species, no growth inhibition was observed in non-target phytoplankton species and organisms (ciliate and zooplankton) at higher trophic levels, which presented an increased species diversity index following the addition of this substance^13^. However, despite these data showing that NQ 2-0 is capable of mitigating only harmful cyanobacterial species without side effects on the aquatic ecosystem, it would be difficult to confidently apply NQ 2-0 to fields having more diverse organisms due to the risk of unexpected side effects on the actual aquatic ecosystem. Therefore, since the understanding of the mechanism about selective algicidal action on cyanobacteria can allow reducing these unexpected side effects, studies on the algicidal mechanism of NQ 2-0 are highly necessary before field application.

Naphthoquinone is a quinone-based, highly redox-active compound that can redox cycle with its semiquinone radical^14^. Quinone-based compounds may have one or more of the following functions: electron transport chain inhibition, oxidative phosphorylation uncoupling, DNA intercalation, reductive alkylation, and free radical generation^15^. Thus, naphthoquinone inhibits photosynthesis by disturbing photosynthetic electron transport in plants^16,17^. However, to date, studies on the algicidal mechanisms of this naphthoquinone substance against phytoplankton species are lacking. Moreover, no studies have attempted to explain its selective algicidal activity. The present study aimed to elucidate the selective algicidal mechanism of NQ 2-0 against cyanobacteria. To address this, variation in photosynthetic activity (oxygen evolution rate and photosynthetic efficiency) of target (*M. aeruginosa*) and non-target (*Cyclotella* sp. and *Selenastrum capricornutum*) species following treatment with NQ 2-0 were examined, and molecular (e.g. reactive oxygen species [ROS], superoxide dismutase [SOD], and catalase [CAT] activities, and malondialdehyde [MDA] contents) and morphological changes in NQ 2-0 treated algal species, allowing to explain that variation in photosynthetic activity can lead to cell death, were also investigated in this study.

## Results

### Difference in the algicidal activity of NQ 2-0 in the presence and absence of light

**Figure 1:**
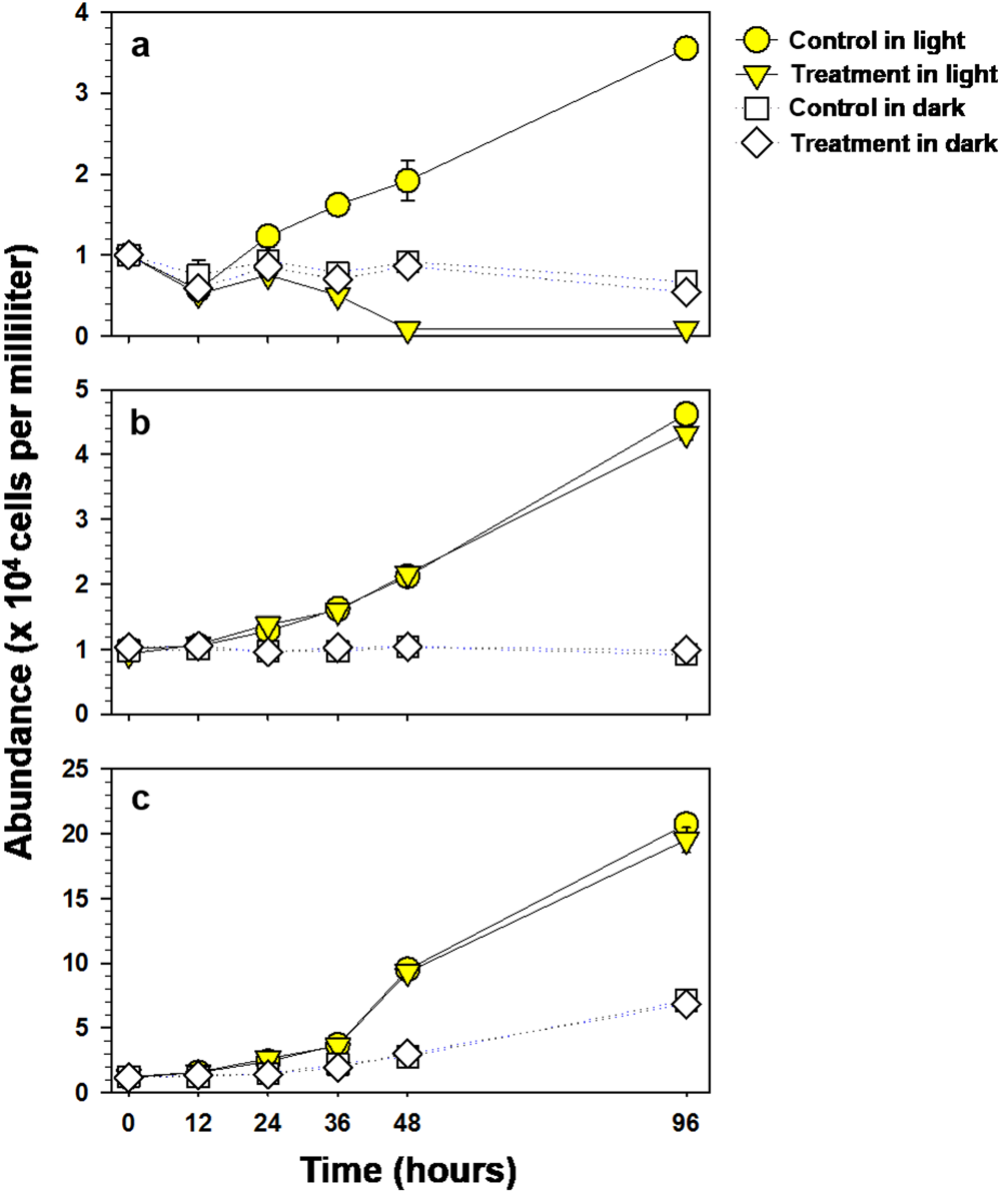
Algicidal activity of NQ 2-0 against *Microcystis aeruginosa* HYK0906-A2 (**a**), *Cyclotella* sp. HYND1404CMZ3 (**b**), and *Selenastrum capricornutum* CCAP278/4 (**c**) according to light and dark conditions.

In the presence of light, the algicidal activity of NQ 2-0 against *M. aeruginosa* was initiated 24 h after the addition of 1 µM NQ 2-0, and reached a maximum at 48 (95.7%) or 96 h (97.4%) (Fig. 1a). Generally, when cultured in the dark, there was no significant difference in the growth of *M. aeruginosa* in the control and 1 µM NQ 2-0 treatment groups, although a low level of algicidal activity (19%) was exhibited after 96 h of treatment. The growth of *Cyclotella* sp. and *S. capricornutum* was generally similar between the control and treatment groups, regardless of the presence or absence of light (Fig. 1b,c).

**Figure 2:**
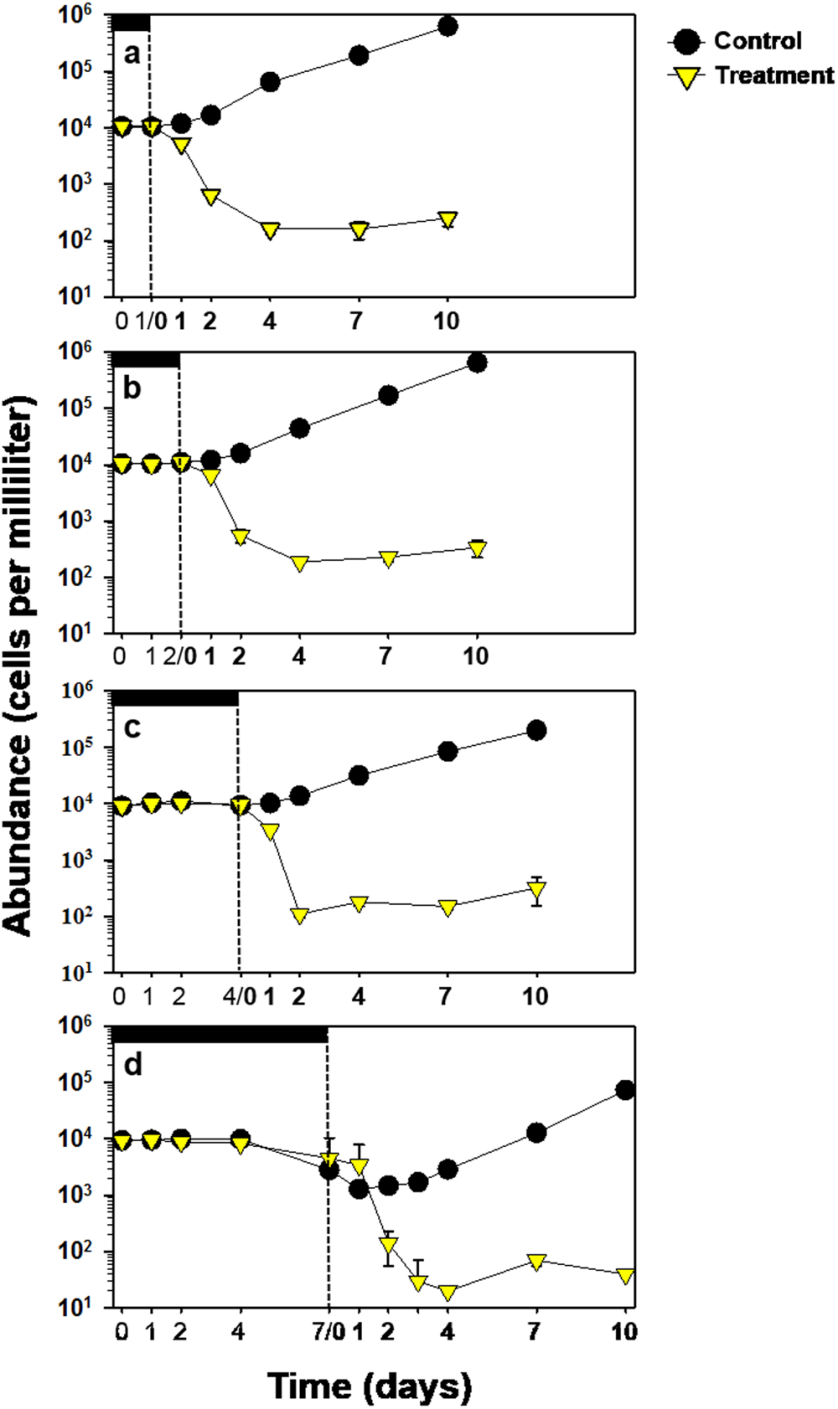
Algicidal activity of NQ 2-0 against *Microcystis aeruginosa* in the presence of light after incubation in the dark for 0 day (**a**), 1 day (**b**), 2 days (**c**), 4 days (**d**), and 7 days (**e**). The vertical dashed lines depict the switch from dark to light.

To better understand the role of light in the algicidal activity on *M. aeruginosa*, this cyanobacterium was incubated for 1, 2, 4, and 7 days in the dark after addition of 1 µM NQ 2-0, and then exposed to a light/dark cycle (Fig. 2). Notably, there was no significant difference in *M. aeruginosa* growth between the control and treatment groups under dark condition. However, regardless of the duration of the dark incubation periods, algicidal activity against *M. aeruginosa* was shown within 24 h after light exposure, and cyanobacterium cells treated with NQ 2-0 were largely lysed at 48 or 96 h (>95%), as well as previous experiment which was conducted under normal condition (Figs 1 and 2).

### Effect of NQ 2-0 on oxygen evolution in photosynthesis

**Figure 3:**
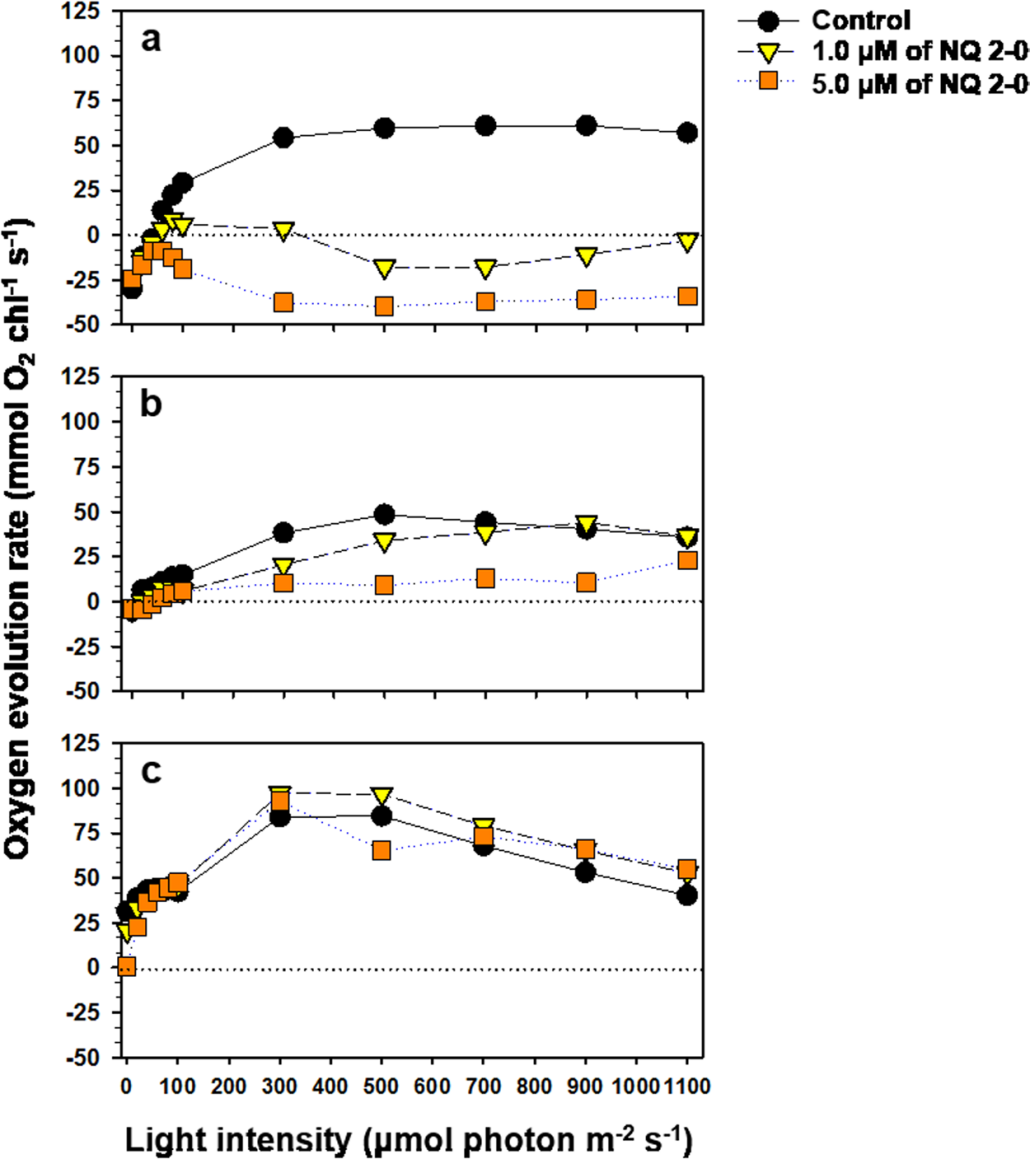
Change in the oxygen evolution rate of *Microcystis aeruginosa* (**a**), *Cyclotella* sp. (**b**), and *Selenastrum capricornutum* (**c**) following treatment with two different concentration (1 and 5 µM) of NQ 2-0.

The oxygen evolution rate of *M. aeruginosa* in the control group increased with ascending light intensity and plateaued at ≥300 μmol photons·m^−2^·s^−1^ (Fig. 3a). Following NQ treatments, these rates were generally lower than those of the control, and appeared to be lower depending on the increase in NQ 2-0 concentration. Moreover, treatment with 1 and 5 µM NQ 2-0 resulted in negative oxygen evolution rates at ≥500 and ≥0 μmol photons·m^−2^·s^−1^, respectively. Similarly, the oxygen evolution rates of *Cyclotella* sp. following NQ 2-0 treatment were lower than those of the control (Fig. 3b). In contrast to the response in *M. aeruginosa*, *Cyclotella* sp. exhibited a consistent positive value up to 1100 μmol photons·m^−2^·s^−1^, even though treatment with 5 µM had a negative value up to 40 μmol photons·m^−2^·s^−1^. The oxygen evolution rates of *S. capricornutum* in control and NQ treatments were mostly similar (Fig. 3c).

### Effects of NQ 2-0 on Photosystem II efficiency (*F*_v_/*F*_m_)

**Figure 4:**
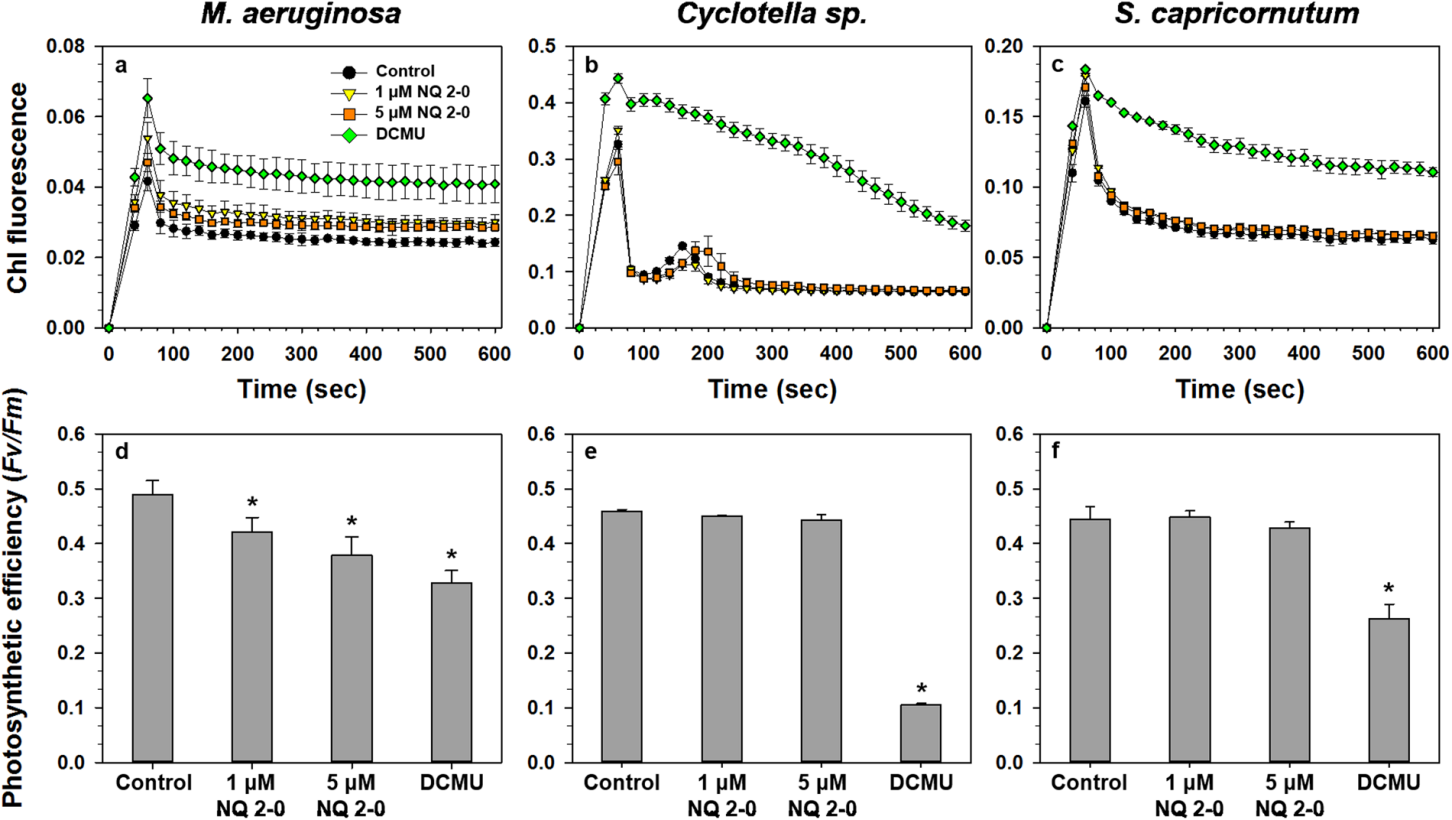
Change in chlorophyll (Chl) fluorescence (**a**–**c**) and Photosystem II efficiency (**d**–**f**) of *Microcystis aeruginosa* (**a** and **d**), *Cyclotella* sp. (**b** and **e**), and *Selenastrum capricornutum* (**c** and **f**) following treatment with two different concentration (1 and 5 µM) of NQ 2-0 and 3-(3, 4-dichlorophenyl)-1, 1-dimethylurea (DCMU). *Represents a significant difference between the control and treatment groups at the *p* ≤ 0.05 level.

To obtain more solid evidence to support the algicidal mechanisms of NQ 2-0, we investigated the photosynthetic efficiency of *M. aeruginosa*, and variations in photosystem II (PSII) were determined based on the addition of NQ 2-0. Chlorophyll (Chl) fluorescence of *M. aeruginosa* was generally higher (1.11–1.29-fold increase) with NQ 2-0 treatment than with the control, and treatment at a higher concentration (5 µM) resulted in slightly lower Chl fluorescence than treatment at a lower concentration (1 µM) (Fig. 4a). PSII efficiency (*F*_v_/*F*_m_) in *M. aeruginosa* was significantly (*p* ≤ 0.05) decreased following the addition of NQ 2-0, and higher concentration treatment had slightly lower efficiency than lower concentration treatment even though there was no significant difference between higher and lower concentration treatments (*p* > 0.05); PSII efficiencies in the control and 1 and 5 µM treatments groups were 0.489, 0.412 and 0.379, respectively (Fig. 4d). There was no clear variation in Chl fluorescence and PSII efficiency (*F*_v_/*F*_m_) of *Cyclotella* sp. and *S. capricornutum* following the addition of NQ 2-0 (Fig. 4b,c,e,f). Treatment with 5 µM 3-(3,4-dichlorophenyl)-1,1-dimethylurea (DCMU) increased the Chl fluorescence of *M. aeruginosa*, *Cyclotella* sp., and *S. capricornutum* to 1.47–1.73, 2.64–4.79, and 1.57–1.98-times that of the control, respectively (Fig. 4a–c), and the photosynthetic efficiencies of *M. aeruginosa*, *Cyclotella* sp., and *S. capricornutum* were 2.61-. 9.43-, and 3.80-times lower than each control (Fig. 4e,f).

### Effects of NQ 2-0 on ROS levels

**Figure 5:**
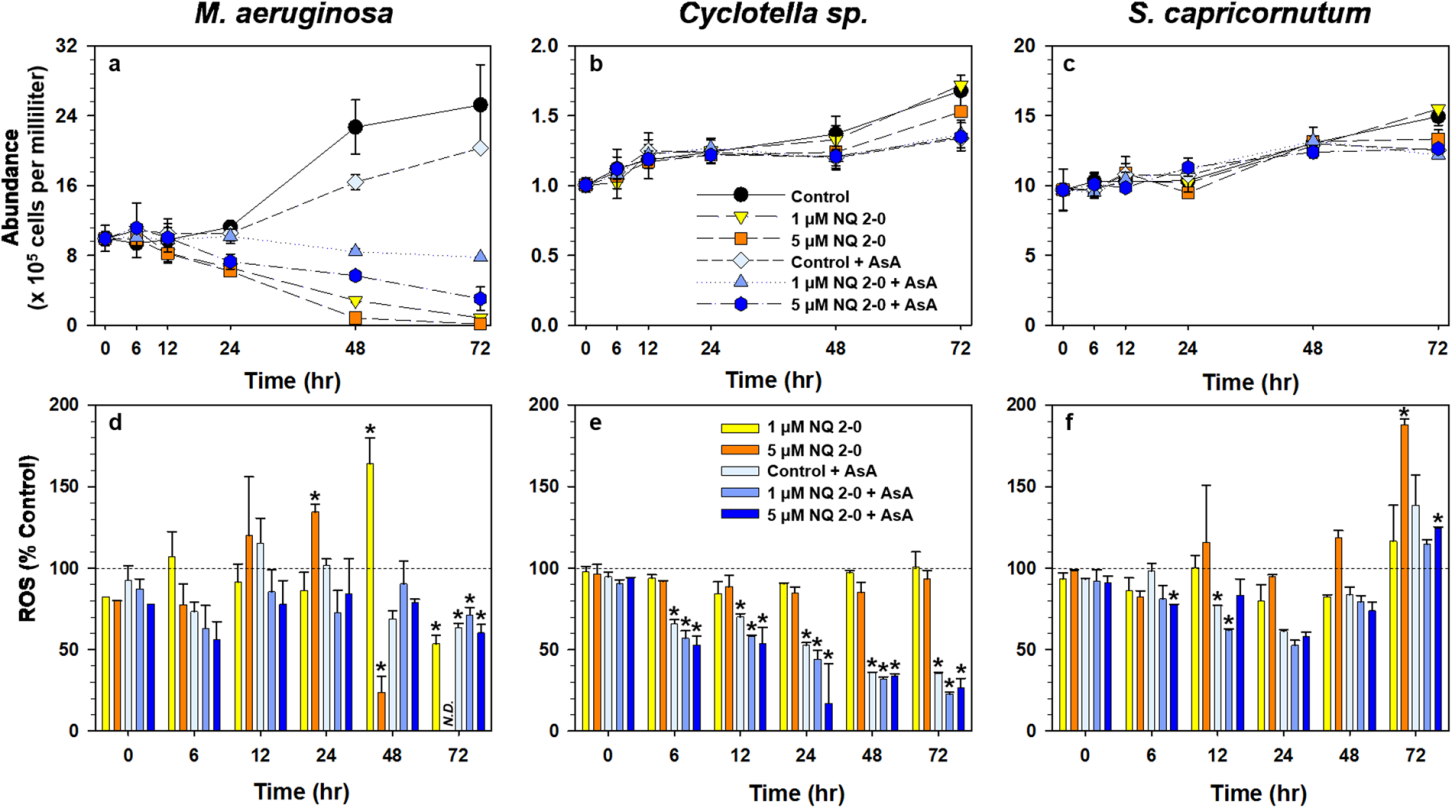
Change in abundance (**a**–**c**) and reactive oxygen species (ROS) generation (**d**–**f)** in *Microcystis aeruginosa* (**a** and **d**), *Cyclotella* sp. (**b** and **e**), and *Selenastrum capricornutum* (**c** and **f**) following treatment with two different concentration (1 and 5 µM) of NQ 2-0 and ascorbic acid (AsA). *Represents a significant difference between the control and treatment groups at the *p* ≤ 0.05 level.

NQ 2-0 at both 1 and 5 µM exhibited up to 99% algicidal activity in *M. aeruginosa* within 72 h, and the biggest reduction of *M. aeruginosa* cells was observed between 24 and 48 h (Fig. 5a). Treatment with 1 µM NQ 2-0 increased the level of ROS to >160% of the control at 48 h and decreased it at 72 h (Fig. 5d). Treatment with 5 µM NQ 2-0 increased the level of ROS to 134% of the control at 24 h, and decreased it at 48 h; ROS levels could not be measured at 72 h because of the low cell density. The growth of *M. aeruginosa* following treatment with ascorbic acid (AsA) was inhibited (19.6%), but the algicidal activity of NQ 2-0 on *M. aeruginosa* was clearly reduced when this cyanobacterium was treated with NQ 2-0 plus AsA; those activities with 1 and 5 µM NQ 2-0 treatment in combination with AsA were 69.10 and 87.92%, respectively. The level of ROS with the NQ 2-0 plus AsA treatment was generally lower than that with the control and NQ 2-0 treatments (Fig. 5d).

The algicidal activity on *Cyclotella* sp. was not observed with 1 µM NQ 2-0 treatment until 72 h. The average abundance of this diatom in 5 µM NQ 2-0 treatment was slightly lower (8.93%) than control, but these difference was not significant (*p* > 0.05) (Fig. 5b). The levels of ROS with 1 and 5 µM NQ 2-0 treatments were generally similar to those with the control (Fig. 5e). The cell growth of AsA–treated *Cyclotella* sp. was hampered compared with the control. Compared with AsA treatment, no algicidal activity was observed following treatment with NQ 2-0 plus AsA (Fig. 5b). In addition, ROS generation following treatment with AsA and NQ 2-0 plus AsA was lower compared with the control during an experiment (0–72 h) (Fig. 5e). Treatment with 1 µM NQ 2-0 presented no algicidal activity, and did not affect the level of ROS in *S. capricornutum* (Fig. 5c,f). By comparison, following treatment with 5 µM NQ 2-0, a 10.70% algicidal activity was observed at 72 h, and the level of ROS was significantly higher (188%) than that of the control at 72 h. As observed for other species, the growth of *S. capricornutum* was inhibited following treatment with AsA; a 16.05% reduction in cell abundance was observed at 72 h with AsA treatment (Fig. 5c). However, there was no additional algicidal activity with the addition of NQ 2-0 at 1 µM and 5 µM to AsA treatment. ROS levels in *S. capricornutum* treated with AsA were generally elevated; ROS levels with AsA treatment (138%) and 1 µM NQ 2-0 plus AsA treatment (124%) were higher than those of the control at 72 h, although the difference was not significant (*p* > 0.05). By comparison, following treatment with 5 µM NQ 2-0 plus AsA (124%), ROS levels were significantly (*p* ≤ 0.05) higher than the control at 72 h; however, these were clearly lower than those with 5 µM NQ 2-0 treatment (33.84%), indicating that AsA reduced the level of ROS.

### Changes in antioxidant enzyme activity and lipid peroxidation with the addition of NQ 2-0

**Figure 6:**
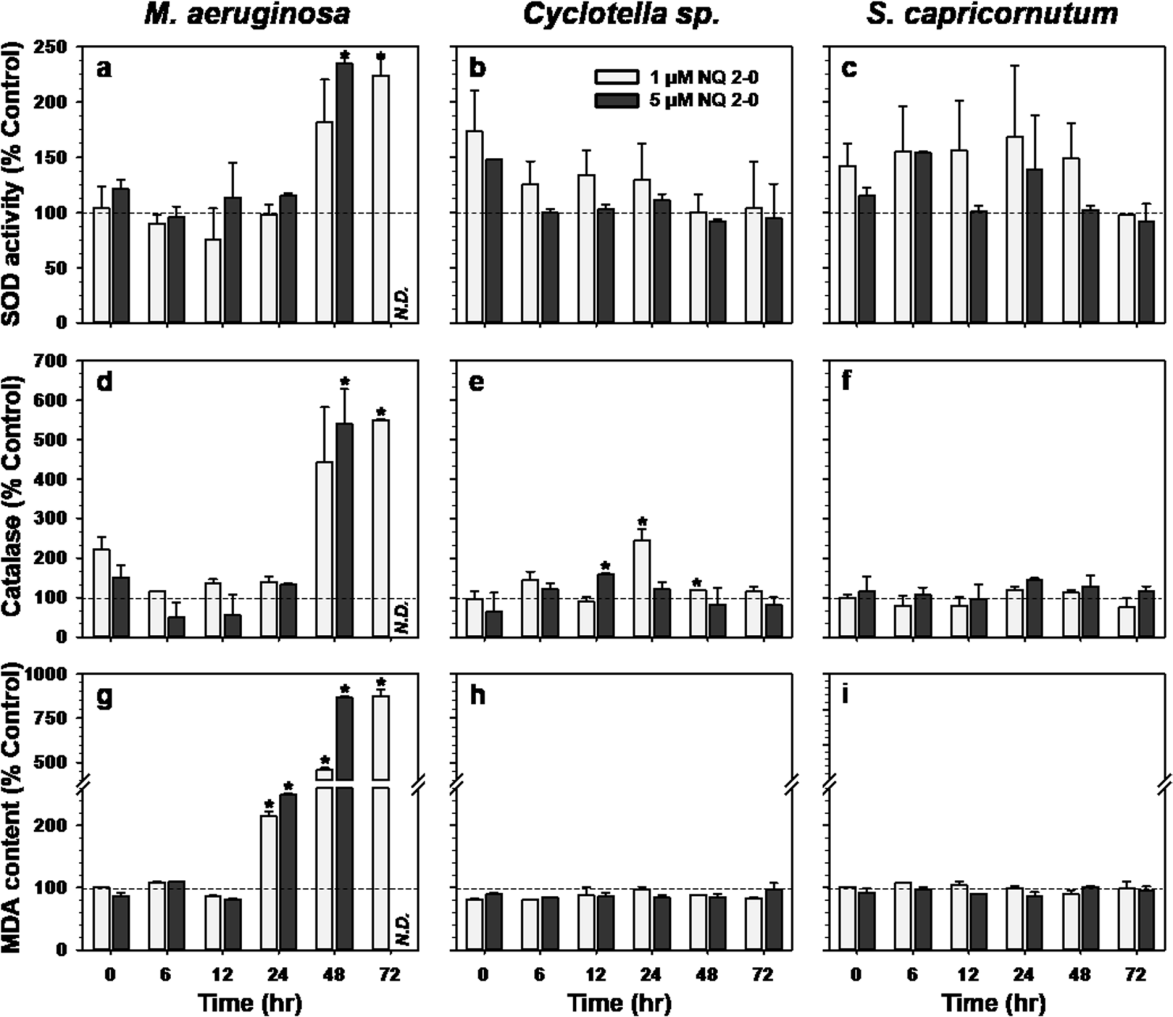
Change in the activities of superoxide dismutase (SOD) (**a**–**c**), catalase (**d**–**f**), and malondialdehyde (MDA) (**g**–**i**) in *Microcystis aeruginosa* (**a**,**d** and **g**), *Cyclotella* sp. (**b**,**e** and **h**), and *Selenastrum capricornutum* (**c**,**f** and **i**) following treatment with two different concentration (1 and 5 µM) of NQ 2-0. *Represents a significant difference between the control and treatment groups at the *p* ≤ 0.05 level.

The SOD activity of *M. aeruginosa* was significantly enhanced following treatment with 1 µM NQ 2-0 (up to 224% of control activity at 72 h; Fig. 6a). At 5 µM NQ 2-0, SOD activity increased to 235% compared with the control at 48 h, but was undetectable due to low cell density at 72 h. The CAT activity of *M. aeruginosa* with 1 µM NQ 2-0 treatment increased to 549% of control activity at 72 h (Fig. 6d). Following 5 µM NQ 2-0 treatment, CAT activity increased to 539% compared with the control at 48 h, and could not be measured because of low cell density at 72 h.

There was no significant variation in SOD activity of *Cyclotella* sp. following the addition of NQ 2-0. By comparison, CAT activity was enhanced by 1 µM NQ 2-0 treatment (to 244 and 118% of control activity at 24 and 48 h, respectively; Fig. 6b,e), and following treatment with 5 µM NQ 2-0, it increased to 158% of control activity at 12 h. Finally, the SOD and CAT activities of *S. capricornutum* were not significantly affected by NQ 2-0 treatment (Fig. 6b,e).

The extent of lipid peroxidation was assessed by determination of MDA levels. MDA levels in *M. aeruginosa* treated with NQ 2-0 were significantly higher than those of the control from 24 h to 72 h, and there was a greater increase in MDA levels with NQ 2-0 at higher concentration; MDA levels following treatment with 1 and 5 µM NQ 2-0 were 214 and 249% at 24 h, and 459 and 866% at 48 h, respectively (Fig. 6g). At 72 h, MDA levels with 1 µM NQ 2-0 treatment were 874%, whereas they could not be measured with 5 µM NQ 2-0 treatment due to the extremely low cell density. In *Cyclotella* sp. and *S. capricornutum*, MDA levels were not significantly affected by the addition of NQ 2-0 (Fig. 6h,i).

### Ultrastructural changes of algae induced by NQ 2-0 treatment

**Figure 7:**
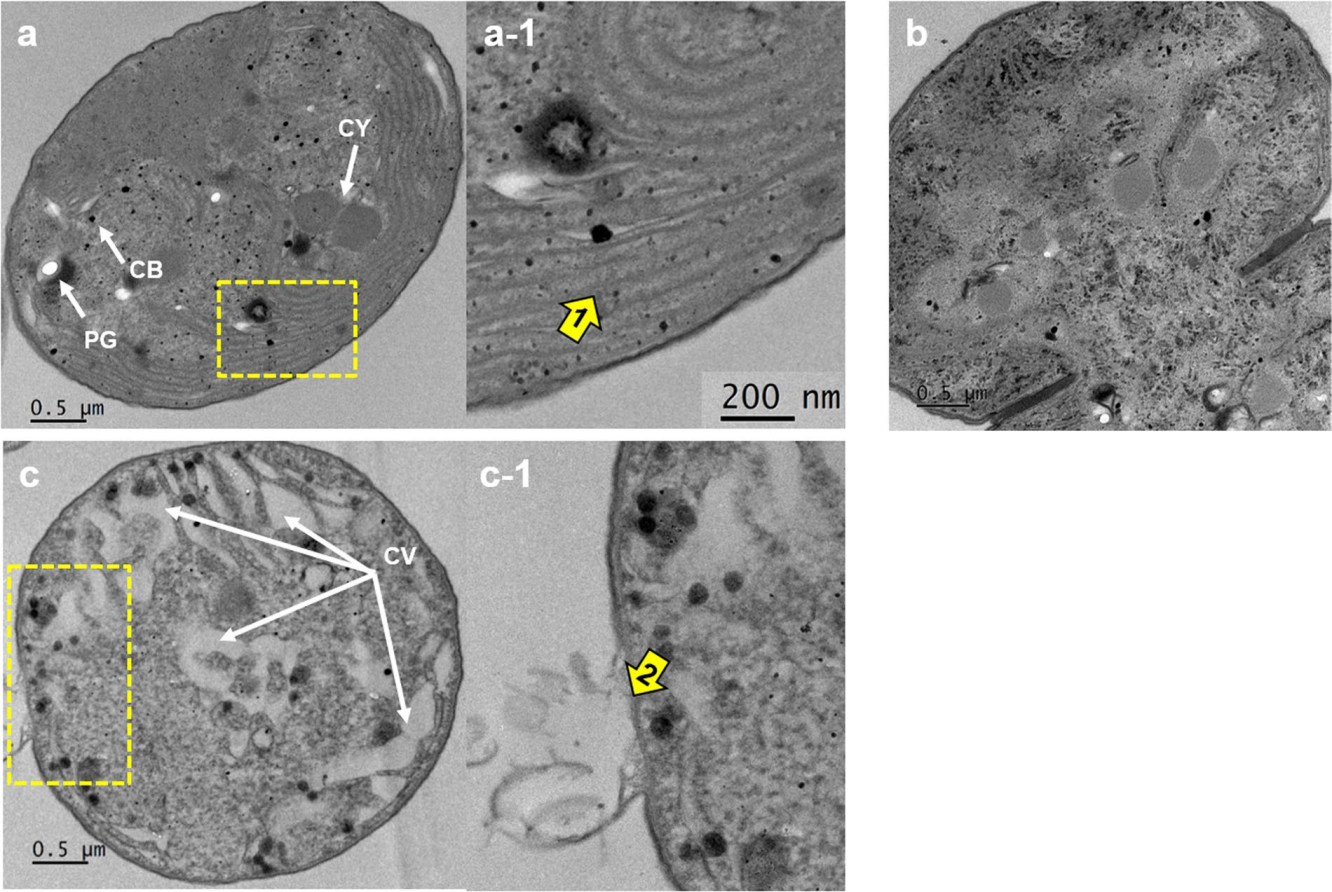
Transmission electron microscopic images of *Microcystis aeruginosa* at 0 h (**a** and a–1), 12 h (**b**), and 24 h (**c** and c–1) after the addition of NQ 2-0 at a final concentration of 1 µM. a–1 and c–1 are enlarged views of the blue windows in a and c, respectively. CB, carboxysomes; CV, cytoplasmic vacuolation; CY, cyanophycin granules; PG, polyphosphate granules, 1; thylakoid membrane (indicated by an arrow), 2; the disintegration of cellular membrane (indicated by an arrow).

To better observe the changes in ultrastructure of *M. aeruginosa* cells following NQ 2-0 treatment, transmission electron microscopy (TEM) was used. Well-defined thylakoid membranes and dense cytoplasm were observed before treatment (Fig. 7a,a-1); however, thylakoid membranes disappeared at 12 h after addition of NQ 2-0 (Fig. 7b). In addition, numerous cytoplasmic vacuolations and the disintegration of cellular membrane were observed at 24 h (Fig. 7c,c-1).

## Discussion

Studying algicidal mechanisms can help to determine whether developed chemical substances have selective algicidal activity against target algal species, thus, reducing side effects on non-target organisms. NQ 2-0 exhibits selective algicidal activity against cyanobacteria. However, we were unable to explain why this chemical substance displayed selective algicidal activity due to a lack of evidence for its algicidal mechanisms. In this study, we investigated the algicidal mechanism of NQ 2-0 by comparing molecular and morphological changes in target (*M. aeruginosa*) and non-target (*Cyclotella* sp. and *S. capricornutum*) organisms, following treatment with NQ 2-0. As a first step, differences in the algicidal activity of NQ 2-0 depending on the presence and absence of light were investigated, since evidence suggests that naphthoquinone can inhibit plant photosynthesis by disturbing the photosynthetic electron transport system^16,17^. Consequently, NQ 2-0 demonstrated a strong algicidal activity only against *M. aeruginosa* under light conditions (>90%) (Fig. 1). Interestingly, under dark conditions, no algicidal activity was observed against target cyanobacterium. Moreover, regardless of the duration of the dark periods, algicidal activity was induced within 24 h of exposing NQ-treated *M. aeruginosa* cells to light, and most of treated cyanobacterium cell was lysed at 48 or 96 h (Fig. 2). These findings suggest that the algicidal activity of NQ 2-0 is strongly associated with light, which might be a key trigger for the induction of algicidal activity.

To resolve the association between light and algicidal activity against *M. aeruginosa*, we first examined the effect of NQ 2-0 on photosynthetic capacity by measuring the rate of oxygen evolution (Fig. 3). In the presence of NQ 2-0, the oxygen evolution rate of *M. aeruginosa* decreased and became negative, indicating that cells could not produce oxygen normally and instead consumed oxygen. Under stress, the consumption of oxygen by algae increases due to a higher respiration rate^18^. Thus, the increased oxygen consumption indicates that NQ 2-0 may act as a stressor of *M. aeruginosa*. By comparison, non-target species exhibited distinct variation in the oxygen rate after the addition of NQ 2-0. The oxygen rate of *S. capricornutum* in response to NQ 2-0 treatment was generally increased compared with the control. In addition, even although the oxygen evolution rate of *Cyclotella* sp. decreased following the addition of NQ 2-0, the rate remained positive, indicating that *Cyclotella* sp. were still able to photosynthesize. These findings indicate that stress caused by NQ 2-0 on two non-target eukaryotic algae may be less than that on the target cyanobacterium.

Based on previous studies, naphthoquinone-based materials, such as halogenated naphthoquinones, are known to inhibit photosynthesis by blocking electron transfer from the photosynthetic electron transport system^16^. In this study, to investigate whether NQ 2-0 can block electron transfer from the photosynthetic electron transport system, we examined changes in PSII efficiency (*Fv*/*Fm*) following the addition of NQ 2-0, and compared the results with those obtained following the addition of DCMU, which is capable of binding to the Q site of PSII and blocking energy transfer to the photosynthetic electron transport system to inhibit photosynthesis^19^. Interestingly, irrespective of target and non-target species, DCMU has significantly (*p* ≤ 0.05) reduced the PSII efficiency of all species (Fig. 4). Conversely, NQ 2-0 showed a different pattern depending on algal species; a clear and dose-dependent reduction in PSII efficiency was observed following the treatment of *M. aeruginosa* with NQ 2-0; however, there was no significant reduction of PSII efficiency in *Cyclotella* sp. or *S. capricornutum*. Given these findings, NQ 2-0 may selectively block electron transfer from the photosynthetic electron transport system in the target cyanobacterium, *M. aeruginosa*, resulting in reduction of PSII efficiency, even though it is difficult to explain why NQ 2-0 can inhibit the photosynthesis activity in *M. aeruginosa*, selectively, using the present data, and this reduced photosynthetic efficiency may induce to decrease the oxygen rate of *M. aeruginosa* which was treated with NQ 2-0.

Based on previous findings, the increase in oxygen consumption and the decrease in photosynthetic efficiency indicate that the photosynthetic system is under stress^20,21^. Thus, reduction in the oxygen rate and PSII efficiency of *M. aeruginosa* following addition of NQ 2-0 implies that this NQ compound can induce a stress in the photosynthetic system of this cyanobacterium. To determine whether this stress can associate with algicidal activity, the level of ROS, as an indicator of cellular stress, was measured^22^. ROS levels were significantly (*p* ≤ 0.05) enhanced in *M. aeruginosa* cultures treated with NQ 2-0 at 24 h (5 µM NQ 2-0) and 48 h (1 µM NQ 2-0) when the cell abundance was dramatically decreased (Fig. 5a,d). By comparison, in *Cyclotella* sp. and *S. capricornutum*, whose growth was not markedly affected by the addition of NQ 2-0, ROS levels were generally similar to those of the control (Fig. 5b,c,e and f). These findings suggest that NQ 2-0 may increase the level of ROS only in the target species, *M. aeruginosa*, and this increase may lead to cell death. However, based on our data, there were conflicting results; at 48 h, the ROS level in *M. aeruginosa* treated with 5 µM NQ 2-0 was significantly lower than that of the control, even though large numbers of cells were lysed. ROS generated in the cell react with lipids and cause lipid peroxidation, which can be measured using MDA as a marker^23^. In *M. aeruginosa*, the MDA level significantly (*p* ≤ 0.05) increased from 24 h of NQ 2-0 treatment (Fig. 6g) indicating an increase in lipid peroxidation caused by accumulating ROS. Interestingly, at 48 h, the level of MDA following treatment with the higher dose (5 µM NQ 2-0) was 1.89-times higher than that following treatment with the lower dose (1 µM NQ 2-0), even though the ROS level with 5 µM NQ 2-0 was 0.15-times lower than that with 1 µM NQ 2-0. Therefore, the lower ROS level in *M. aeruginosa* treated with 5 µM NQ 2-0 at 48 h is likely due to the interaction between ROS and lipids. To obtain more solid evidence of linkage between levels of ROS and cell death, we used AsA, a ROS scavenger^24^, to determine whether the reduced ROS level following AsA addition would decrease the algicidal activity of NQ 2-0 on *M. aeruginosa*. Based on our data, ROS levels in algal cells were reduced following treatment with NQ 2-0 plus AsA, compared with NQ 2-0 alone (Fig. 5d). Interestingly, although AsA alone demonstrated slight algicidal activity, it significantly (*p* ≤ 0.05) reduced the algicidal activity of NQ 2-0 against *M. aeruginosa* (Fig. 5a). These findings suggest that the increased ROS levels may account for cell death of *M. aeruginosa*.

The cellular structures of treated *M. aeruginosa* were observed by light microscopy and TEM (Figs 7 and S1). After NQ 2-0 treatment (1 μM), chlorosis (bleaching) of the cells and degradation of intracellular structures were observed at 24 h when algicidal activity against target cyanobacterium was generally appeared (Figs 1 and S1). Interestingly, in TEM observation, thylakoid and cellular membranes in *M. aeruginosa* were disintegrated at 12 h and 24 h after addition of NQ 2-0, respectively (Fig. 7b). Based on previous findings, ROS can cause lipid peroxidation in membranes, including thylakoid and cellular membrane, resulting in destruction of those membranes^23^. In our results, after 6 h of NQ 2-0 treatments, levels of ROS and MDA in *M. aeruginosa* was enhanced, and reached the highest at between 24 and 72 h (Figs 5d and 6g). Thus, these disintegrated membranes in *M. aeruginosa* is likely result of increase in lipid peroxidation caused by ROS generation following NQ 2-0 treatment. Interestingly, cytoplasmic vacuolation which is a feature regarded to be a cytological hallmark of paraptosis^25^ was observed at 24 h (Fig. 7c). Cytoplasmic vacuolation in microalgal species was generally appeared during programmed cell death^26,27^. These results suggest that increased stress level (e.g. ROS and lipid peroxidation) in *M. aeruginosa* caused by NQ 2-0 treatment may be able to induce programmed cell death.

NQ 2-0 at the higher concentration (5 µM) exhibited a slight algicidal activity on *Cyclotella* sp. and *S. capricornutum*, even though its activity (8.93–10.07%) on non-target species was much lower than that on target species (>99%). Interestingly, the level of ROS in *S. capricornutum* cultured treated with 5 µM NQ 2-0 was significantly enhanced at 72 h when a slight algicidal activity was observed. Although there was no significant enhancement of the ROS level in *Cyclotella* sp. following NQ 2-0 treatment, the oxygen rate of *Cyclotella* sp. treated with this NQ compound was slightly decreased, implying that NQ 2-0 might cause a stress for this diatom. These results indicate that higher concentrations (5 µM) of NQ 2-0 can act as a stressor of non-target species, as well as target *M. aeruginosa*, even though the extent of stress level by higher concentration NQ 2-0 seemed to be different between target and non-target species. The following question was raised from this finding; if NQ 2-0 can increase ROS levels in target and non-target species, how do non-target species, unlike target species, reduce the algicidal activity of this algicide. Previous studies have shown that antioxidant enzymes can remove excess ROS in cells^28^. SOD and CAT activity in *M. aeruginosa* was enhanced after 48 h, while the ROS level increased within 24 h (Figs 5d and 6a,d). Moreover, the MDA level which can be indicator of damage level in cell was significantly enhanced at 24 h (Fig. 6g). It can be speculated that *M. aeruginosa* did not respond quickly to the initial increase in ROS, and therefore few cells could survive, whereas SOD activity in *Cyclotella* sp. and *S. capricornutum* increased immediately after exposure to NQ 2-0. Thus, non-target species may have responded fast enough to quench superoxides to protect cells and minimize the algicidal activity of NQ 2-0 (Fig. 5b,c). Based on the previous finding, different pigment can exhibit different antioxidant activities and profiles, which are capable of detoxifying NQ 2-0^29^. Interestingly, chlorophyll pigment composition between cyanobacteria and eukaryotic algae considerably differs; cyanobacteria possess Chl *a* and phycobilin pigments^30^; green algae possess Chl *a* and *b*, β-carotene, and various xanthophylls^31,32^; and diatoms possess Chl *a* and *c*, β-carotene, fucoxanthin, diatoxanthin, and diadinoxanthin^33^. Therefore, it is likely that the particular algicidal activity of NQ 2-0 against cyanobacteria may be due to differences in the pigments, which may differentially quench toxic substances such as ROS generated by NQ 2-0.

**Figure 8:**
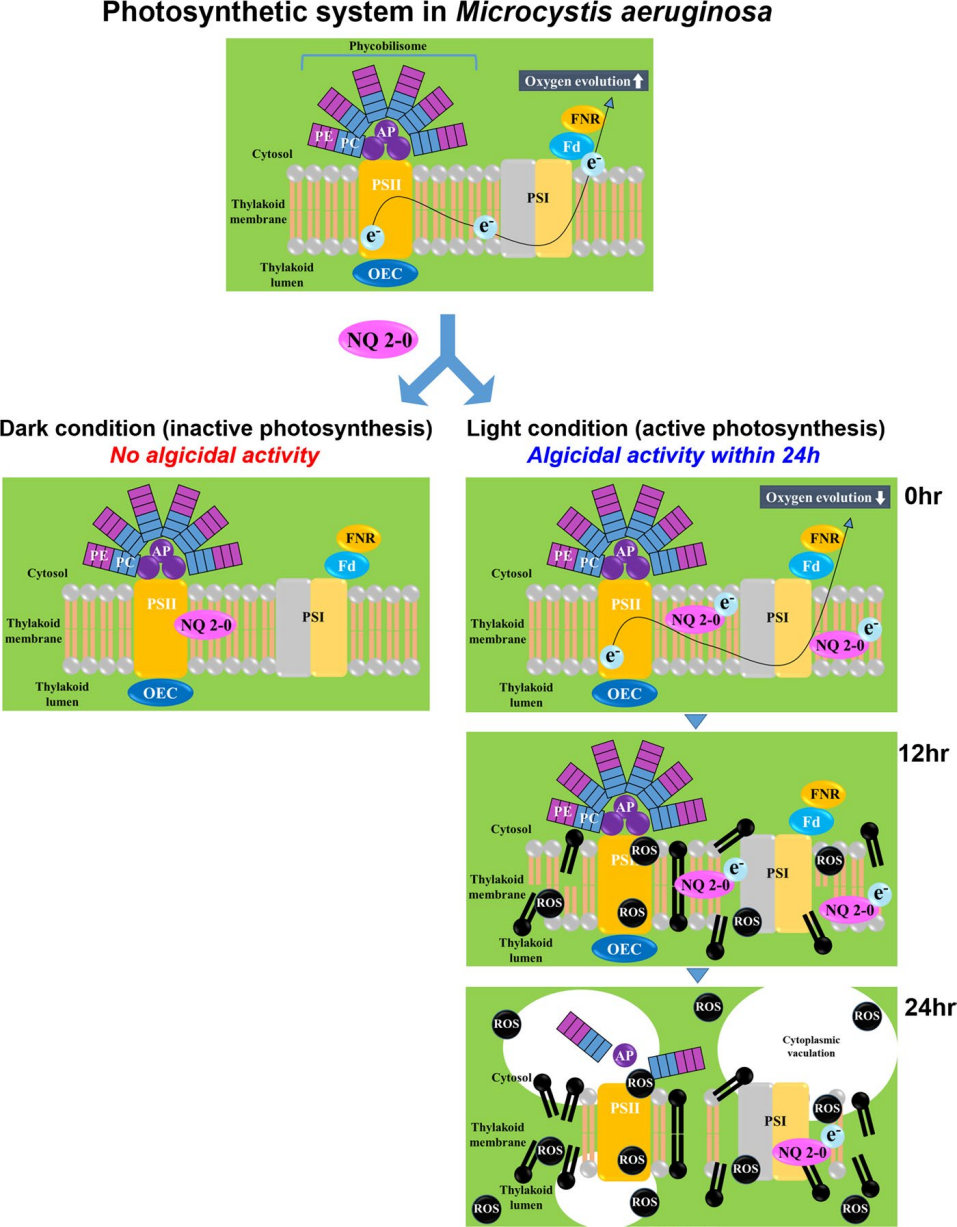
Schematic diagram for the algicidal mechanisms of NQ 2-0 against *Microcystis aeruginosa*.

This study has shown, for the first time, how this algicide can have a selective algicidal activity against only target cyanobacterium *M. aeruginosa*. NQ 2-0 showed selective algicidal activity against cyanobacterium *M. aeruginosa*, and this activity was strongly light-dependent. This findings indicate that this NQ compound may affect cyanobacterial photosynthetic system, selectively. To better understand this, we investigated the effect of this NQ compound on photosynthetic system in target and non-target species. Consequently, NQ 2-0 selectively caused a stress for photosynthetic activity of *M. aeruginosa* throughout blocking electron transfer from the photosynthetic electron transport system, resulting in reduction of PSII efficiency, and this stress leaded to an increase in ROS levels, which causes lipid peroxidation in cell membranes (Fig. 8). In addition, throughout treatment with NQ 2-0 plus AsA, we can conclude that increased ROS levels following addition of NQ 2-0 may be directly associated with cell death. Besides, in microscopic observations, disintegrated thylakoid membrane was detected in *M. aeruginosa* treated with NQ 2-0. This disintegration is likely result of increase in lipid peroxidation caused by ROS generation following NQ 2-0 treatment. In addition, cytoplasmic vacuolation, resulting in paraptosis and programmed cell death, was also observed in *M. aeruginosa* cells which were treated with NQ 2-0. Lastly, compared to non-target eukaryotic cells, relatively late-response in *M. aeruginosa* to reduce the increased ROS level was also thought to contribute on enhancement of algicidal activity against this cyanobacterium. However, it is still unclear how NQ 2-0 can block electron transfer from the photosynthetic electron transport system in cyanobacteria, selectively. Based on our previous study, a number of NQ compound was developed, but NQ 2-0 is the only compound, showing a selective algicidal activity against cyanobacteria. Therefore, this selectivity may be caused by specific structure of NQ 2-0. Therefore, in future, additional research, such as characterization of the chemical structure which can selectively kill target cyanobacterium, is needed to further elucidate the selective algicidal mechanisms in future.

## Materials and Methods

### Algal cultures

Cultures of *M. aeruginosa* HYK0906-A2 (Cyanophyceae), *Cyclotella* sp. HYND1404CMZ3 (Bacillariophyceae), and *S. capricornutum* CCAP278/4 (Chlorophyceae), which belong to different taxonomic classes, were used in this study. These cultures were obtained from the National Research Laboratory for Water Environmental Ecology and Restoration of Hanyang University (Seoul, South Korea) and the Culture Collection of Algae and Protozoa (CCAP; United Kingdom). All cultures were maintained at 15 °C (*Cyclotella* sp) and 20 °C (*M. aeruginosa* and *S. capricornutum*) in cyanobacteria medium for *M. aeruginosa*^34^, diatom medium for *Cyclotella* sp.^35^, and in a combination of *Euglena gracilis* medium and Jaworski’s medium (1:1) for *S. capricornutum*^36^ under cool-white fluorescent lamps (photon flux of 50 μmol photons·m^−2^·s^−1^) on a 12-hour light:12-hour dark photoperiod.

### Variation in the algicidal activity of NQ 2-0 in the presence and absence of light

Duplicate cultures with an initial cell density of 1 × 10^4^ cells mL^−1^ were treated with the algicidal agent NQ 2-0 at a final concentration of 1 μM. To investigate the association between light and the algicidal activity of NQ 2-0, two experiments were carried out. In the first experiment, NQ-treated and control cultures were incubated in the presence (50 μmol photons·m^−2^·s^−1^, 12-hour light:12-hour dark photoperiod) and absence of light for 4 days. Subsamples used to count algal cells were taken from duplicate flasks at 0, 12, 24, 48, and 96 h. In the second experiments, cultures were incubated under dark conditions for various time periods (1, 2, 4, and 7 days) and then exposed to light. Subsamples were collected at 1–3-day intervals during the dark and light periods. All subsamples were fixed with Lugol’s solution at a final concentration of 2%, transferred onto a Sedgewick-Rafter chamber, and algal cells were counted at 200× magnification using a light microscope (Olympus, Japan). Algicidal activity (%) was calculated by the following equation: Algicidal activity (%) = (1 − T*t*/C*t*) × 100, where, C and T are the algal cell densities in control and NQ 2-0 treatments, and *t* is the time of the measurement in hours.

### Physiological changes in response to NQ 2-0 treatment

#### Oxygen evolution rate

The oxygen evolution rate was measured using a Clark-type oxygen electrode (Oxygraph; Hansatech Instruments Ltd., England) as described by Kim *et al*.^37^. Briefly, each sample comprising a cell suspension (0.95 mL) with a final Chl concentration of 2 μM, and 0.5 M NaHCO_3_ (0.05 mL) as the carbon source, was loaded into the oxygen electrode chamber and incubated in the dark for 10 min. The oxygen evolution rate was then measured at various light intensities (20, 40, 60, 80, 100, 300, 500, 700, 900, and 1100 μmol photons·m^−2^·s^−1^) for 2 min. During the analysis, the temperature was maintained at 25 °C using a circulating water jacket, and illumination was provided by a slide projector lamp (Osram, Germany).

#### Chlorophyll fluorescence and Photosystem II efficiency (F_v_/F_m_)

To investigate the effect of NQ 2-0 on the photosynthetic machinery, we monitored variations in PS II efficiency (*Fv*/*Fm*) in three cultures after the addition of NQ 2-0 at two different concentrations (1 and 5 µM) by measuring Chl fluorescence. In addition, to determine whether NQ 2-0 negatively affects photosystem II of *M. aeruginosa*, DCMU treatment was used as a positive control; DCMU blocks the Q site in PSII^19^. In this study, Chl fluorescence was measured via a pulse amplitude modulation fluorescence meter (FMS1; Hansatech Instruments Ltd.) following the protocol described by Kim *et al*.^37^. Briefly, prior to measurement of Chl fluorescence, samples containing 1 nM total Chl were dark-adapted at 20 °C for 10 min and were then transferred into a dark chamber. PS II efficiency (maximum quantum efficiency of PSII photochemistry) was calculated by the following equation; *F*_v_/*F*_m_ = *(F*_m_ − *F*_0_)/*F*_m_, where, *F*_0_ is the minimal fluorescence of the dark-adapted sample, determined using a weak measuring beam (below 0.05 μmol photons·m^−2^·s^−1^), which was incapable of electron transfer from the photosynthetic electron transport system; *F*_m_ is the maximal fluorescence of the dark-adapted sample, determined using a saturating pulse (18,000 μmol photons·m^−2^·s^−1^); *F*_v_ is the variable fluorescence (*F*_m_ − *F*_0_).

#### Reactive oxygen species

The levels of ROS were measured with the fluorescent probe 2′, 7′-dichlorofluorescein diacetate (DCFH-DA, Sigma-Aldrich, MO, USA). Each sample (3 × 10^6^ cells) was centrifuged at 6000 *g* for 5 min, the pellet was washed twice with phosphate-buffered saline (PBS), and the cells were incubated with PBS containing 10 μM DCFH-DA at 25 °C for 1 h in the dark. DCFH-DA was removed by centrifugation. The pellets were washed twice with PBS and then resuspended in 200 μL of PBS. Samples were transferred to 96-well plates, and fluorescence intensity was measured at an excitation wavelength of 488 nm and an emission wavelength of 525 nm. To determine whether increased ROS levels due to the addition of NQ 2-0 were associated with cell death, NQ 2-0 was added to cultures with the antioxidant AsA at a final concentration of 1 μM, which degrades ROS, and we investigated the change in algal growth and ROS levels. In addition, algal cultures were treated with the same concentration of AsA (AsA treatment) to more accurately evaluate variations in the algicidal activity of NQ 2-0 and ROS levels in plants treated with NQ 2-0 and AsA, depending on the addition of AsA. Subsamples used to measure cellular abundance and ROS levels were collected 0, 6, 12, 24, 48, and 72 h after the addition of two different concentrations NQ 2-0 and NQ 2-0 plus AsA^38^.

#### Antioxidant enzyme activity

SOD and CAT activities were measured using an OxiSelect™ SOD Activity Assay Kit (Cell Biolabs, Cat No.: STA-340, San Diego, CA, USA) and OxiSelect™ Catalase Activity Assay Kit (Cell Biolabs, Cat No: STA-341), respectively, according to the manufacturer’s protocols. Subsamples were collected 0, 6, 12, 24, 48, and 72 h after the addition of two different concentrations (1 and 5 μM) of NQ 2-0.

#### Malondialdehyde content

To investigated lipid peroxidation by ROS, MDA content was measured using the Lipid Peroxidation (MDA) Assay Kit (Sigma-Aldrich, Cat No: MAK085) according to the manufacturer’s protocols. Subsamples were collected 0, 6, 12, 24, 48, and 72 h after the addition of two different concentrations (1 and 5 μM) of NQ 2-0.

### Morphological and ultrastructural changes in response to NQ 2-0 treatment

Cells were observed under a light microscope (Olympus BX51; Olympus Co., Japan) 0 and 24 h after the addition of NQ 2-0 at a final concentration of 1 μM. A TEM was also used to investigate morphological changes in the intracellular ultrastructure of *M. aeruginosa* cultured with 1 μM NQ 2-0. Briefly, cells were collected 0, 12, and 24 h after the addition of NQ 2-0 and harvested by centrifugation at 6000 × *g* for 5 min. The cells were washed twice with PBS and then fixed in 2.5% glutaraldehyde at 4 °C. The cells were treated with 1% OsO_4_ and 1.5% potassium ferrocyanide in 0.1 M phosphate buffer (pH 7.3) at 4 °C in the dark for 1 h, dehydrated with an ethanol and propylene oxide series, and embedded in Epon 812. After 2 days of polymerization at 70 °C, 70 nm sections were cut with an ultramicrotome (UltraCut-UCT, Leica, Austria) and collected on copper grids (100 mesh). After staining with 2% uranyl acetate (15 min) and lead citrate (5 min), bio-TEM images of the sections were acquired (FEI Tecnai G^2^ Spirit Twin, USA) at 120 kV^39^.

### Statistical analysis

To investigate statistical differences between two groups, *t*-tests (*p* ≤ 0.05) were conducted using SPSS version 8.0 (SPSS, IBM, Chicago, IL, USA).

### Data availability

The datasets generated and analysed during this study that are not included in the published article are available from the corresponding author upon reasonable request.

## Electronic supplementary material

Supplementary Figure S1
