# Supplementary material for: Cyanobacteria-specific algicidal mechanism of bioinspired naphthoquinone derivative, NQ 2-0
Source: Sci Rep. 2018 Aug 2;8:11595. doi: 10.1038/s41598-018-29976-5 (PMC6072754; doi:10.1038/s41598-018-29976-5)

(Original Article)

Cyanobacteria-specific algicidal mechanism of bioinspired naphthoquinone derivative, NQ 2-0

Heon Woo Lee[1,†], Bum Soo Park[1,2,†], Jae-Hyoung Joo[1], Shailesh Kumar Patidar[1], Hye Jeong Choi[1], EonSeon Jin[1,]* and Myung-Soo Han[1,3,]*

[1]Department of Life Science, College of Natural Sciences, Hanyang University, Seoul 04763, S. Korea

[2]Marine Science Institute, University of Texas at Austin, Port Aransas, TX 78373, USA

[3]Research Institute for Natural Sciences, Hanyang University, Seoul 04763, S. Korea

Running title: Selective algicidal mechanisms of NQ 2-0

*Corresponding authors:

E. Jin

Tel: +82-2-2220-2561, Fax: +82-2-2299-2561, E-mail: esjin@hanyang.ac.kr

M-.S. Han

Tel: +82-2-2220-0956, Fax: +82-2-2296-1741, E-mail: hanms@hanyang.ac.kr

[†]The first two authors contributed equally to this work.

Figure S1. Light microscopic images of *Microcystis aeruginosa*, *Cyclotella* sp., and *Selenastrum capricornutum* at 0 (A–C) and 24 h (D–F) after the addition of NQ 2-0 at a final concentration of 1 μM.

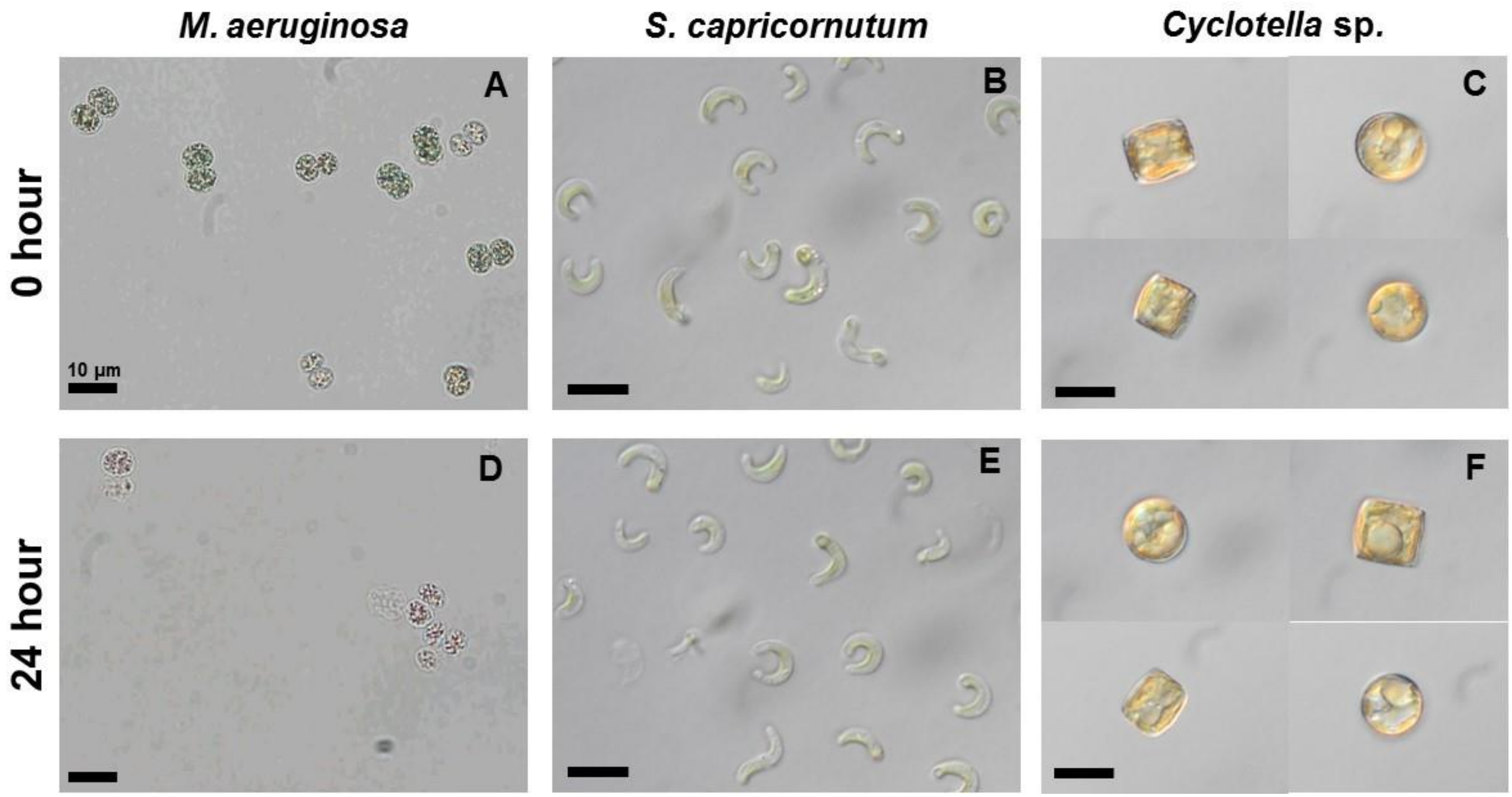

Supplement: Supplementary file 1 — Supplementary Figure S1 [file 41598_2018_29976_MOESM1_ESM.pdf]
